# Interoception and stress

**DOI:** 10.3389/fpsyg.2015.00993

**Published:** 2015-07-20

**Authors:** André Schulz, Claus Vögele

**Affiliations:** Institute for Health and Behaviour, Integrative Research Unit on Social and Individual Development, University of LuxembourgWalferdange, Luxembourg

**Keywords:** chronic stress, HPA axis, interoception, SAM axis, somatization, stress disorders, sympathetic nervous system, symptom perception

## Abstract

Afferent neural signals are continuously transmitted from visceral organs to the brain. Interoception refers to the processing of visceral-afferent neural signals by the central nervous system, which can finally result in the conscious perception of bodily processes. Interoception can, therefore, be described as a prominent example of information processing on the ascending branch of the brain–body axis. Stress responses involve a complex neuro-behavioral cascade, which is elicited when the organism is confronted with a potentially harmful stimulus. As this stress cascade comprises a range of neural and endocrine pathways, stress can be conceptualized as a communication process on the descending branch of the brain–body axis. Interoception and stress are, therefore, associated via the bi-directional transmission of information on the brain–body axis. It could be argued that excessive and/or enduring activation (e.g., by acute or chronic stress) of neural circuits, which are responsible for successful communication on the brain–body axis, induces malfunction and dysregulation of these information processes. As a consequence, interoceptive signal processing may be altered, resulting in physical symptoms contributing to the development and/or maintenance of body-related mental disorders, which are associated with stress. In the current paper, we summarize findings on psychobiological processes underlying acute and chronic stress and their interaction with interoception. While focusing on the role of the physiological stress axes (hypothalamic-pituitary-adrenocortical axis and autonomic nervous system), psychological factors in acute and chronic stress are also discussed. We propose a positive feedback model involving stress (in particular early life or chronic stress, as well as major adverse events), the dysregulation of physiological stress axes, altered perception of bodily sensations, and the generation of physical symptoms, which may in turn facilitate stress.

## Introduction

Interoception, i.e., the perception of bodily processes, plays an important role for symptom generation in body-related mental disorders, such as panic disorder (PD; Ehlers and Breuer, 1996), somatoform disorders (SDs; Bogaerts et al., 2010; Pollatos et al., 2011b), dissociative disorders (Michal et al., 2014; Sedeno et al., 2014; Schulz et al., 2015b), or eating disorders (Pollatos et al., 2008; Herbert and Pollatos, 2014). Stress responses involve a complex neuro-behavioral cascade, which is elicited when the organism is confronted with a potentially harmful stimulus, and includes cognitive (e.g., facilitation of selective attention; Chajut and Algom, 2003), affective (e.g., anxiety or fear; McEwen, 2000), and physiological changes [e.g., activation of the autonomic nervous system (ANS) and the hypothalamic-pituitary-adrenocortical (HPA) axis; Chrousos and Gold, 1992]. Exposure to an acute stressor of limited duration does not normally affect an organism’s health. Nevertheless, the experience of early life stress, chronic stress or major adverse events may represent one of the most important risk factors for mental disorders, cardiovascular diseases, and auto-immune disorders. Previously, it has been proposed that stress alters the central representation of bodily processes (Craig, 2002). Although interoception may be affected differentially across disorders, it is likely that the role of stress in the etiology of these disorders is mediated by interoception. As the knowledge on the interaction of interoception, stress and mental disorders associated with physical symptoms is only fragmentary (e.g., limited to a single interoceptive indicator, to one stress test or one physiological stress axis, or to one mental disorder), the objective of the current review is to summarize and synthesize existing knowledge from both ‘normal’ and ‘dysregulated’ interoception in mental disorders. This review of the existing literature may help to identify yet unanswered questions in this field and our synthesized framework model as developed in this review may stipulate further research in the area.

### Structure of this Review

In the current paper we describe the psychobiology of interoception and stress, as well as a framework to demonstrate why both concepts are interconnected and of immediate relevance for health and disease (see The Ascending and the Descending Branch of the Brain-Body Axis). First, we briefly describe both physiological stress axes with specific attention on processes, which may be relevant for the interaction of interoception and stress (see Physiological Stress Axes). We then review the literature on the relationship between interoception and stress, as well as on the role of interoception in mental disorders, which are associated with both dysregulations of physiological stress axes and physical symptoms (see Method and Results). Before we present the results of our literature search, we summarize methodological approaches to assess facets of interoception, which have been used in the relevant literature (see Methodology to Assess Interoception). The summary of findings on interoception and stress (see Synthesis of Findings on Interoception and Stress) is separated into effects of acute and chronic stress on interoception, and into effects of both physiological stress axes on interoception. The synthesis of findings results in a postulation of a framework model incorporating a positive feedback loop that describes the relationship of (chronic) stress, dysregulation of stress axes, altered perception of bodily sensations and physical symptom. Further, we integrate findings on interoception and stress into existing knowledge on the neurobiology of interoception and speculate on the possibly underlying neuro-endocrine signal circuitry (see Neuroendocrine Pathways). Finally, we identify a number of psychological mechanisms (e.g., attention, learning, intuitive decision making), which are known to be affected by interoception and stress and which may play an additional role in the proposed framework model (Psychobiological Mechanisms Involved in Interoception and Stress).

### Physiological Stress Axes

Physiological systems to provide resources to cope with the confrontation with a potentially harmful stimulus (e.g., elicitation of a fight-or-flight response) can be sub-divided into two partially independent stress axes: (1) The sympatho-adreno-medullary (SAM) axis, including components of the ANS and (2) the HPA axis (for a detailed review: see Chrousos and Gold, 1992; Chrousos, 2009).

The SAM axis represents a heterogeneous network of neural and endocrine functions, which are interconnected to activate sympathetic processes. The release of corticotropin releasing factor (CRF) as neurotransmitter in the locus coeruleus (LC) leads to the activation of medullary centers, which control the sympathetic nervous system. When activated, sympathetic processes may stimulate two mechanisms with different pathways: (a) a neural pathway via ganglia, which innervate effector organs over mainly noradrenergic synapses and (b) an endocrine pathway that elicits the release of catecholamines (e.g., epinephrine and norepinephrine) into the circulation by the adrenal glands. Circulating catecholamines stimulate effector organs via specific adrenergic receptors (e.g., β1-adrenergic receptors at the myocardium). The LC is part of the central noradrenergic system and connected with structures in the limbic system (e.g., amygdalae) and the frontal cortex. Central α2-adrenergic receptors in the noradrenergic system, mainly located in the LC and the nucleus tractus solitarius (NTS), may down-regulate sympathetic activation and thus represent a negative feedback mechanism in the SAM axis (Isaac, 1980).

The HPA axis involves three consecutive stages: when encountering a stressor, (a) CRF is released from the hypothalamus into the blood circuit, which elicits (b) a release of adrenocorticotropic hormone (ACTH) from the pituitary. Circulating ACTH is registered by (c) the adrenal cortex that releases cortisol in humans, or corticosterone in rodents. Cortisol (or corticosterone) may inhibit the production of both CRF and ACTH and thus constitute a negative feedback loop. Receptors for glucocorticoids, such as cortisol, are distributed over all cells in the entire body. Nuclear mineralocorticoid and glucocorticoid receptors may slowly induce changes in gene transcription over a time frame of hours (De Kloet et al., 1998). Furthermore, glucocorticoids may also elicit rapid, non-genomic effects on cells (mediated via membrane receptors) within several minutes (de Kloet et al., 2008; Groeneweg et al., 2011). As every cell is potentially affected by circulating cortisol, it is a challenge for stress research to understand signaling pathways. Cortisol, for example, has been shown to affect metabolism, immune function and various CNS processes, such as sleep and activity, learning and memory, and attention. Nevertheless, CRF and ACTH may also affect CNS processes, as is evident from their role in anxiety and depression (Arborelius et al., 1999; Strohle et al., 2000). Given the interdependence between CRF, ACTH and cortisol via negative feedback mechanisms, all *in vivo* relationships between cortisol and psychological processes cannot be solely attributed to cortisol. However, since the majority of findings on HPA axis activity focuses on cortisol effects, the current review will focus on the latter.

It should be noted that the two axes are not fully independent from each other. For instance, circulating CRF may inhibit central noradrenergic processes, while noradrenergic activity inhibits CRF production in the hypothalamus (Chrousos and Gold, 1992). There is evidence for between-individual specificity in stress response patterns (Fahrenberg and Foerster, 1982), which is assumed to play a role for individuals’ vulnerability for certain disorders (Cohen and Hamrick, 2003). This partial independence of the two stress axes may also imply that a response in one stress axis may not necessarily be associated with the other, depending on individual and environmental factors.

### The Ascending and the Descending Branch of the Brain–Body Axis

The strong relationship between stress and interoception is illustrated by the fact that both processes reflect the communication between the CNS (i.e., the brain) and the periphery (i.e., the body). On the one hand, interoception describes the processing and perception of internal bodily states, which are transmitted from peripheral organs, presumably via afferent nerve fibers, to the brain. Interoception can, therefore, be seen as an example for ascending information on the brain–body axis. On the other hand, stress, can be seen as a prominent example of communication on the brain–body axis in descending direction (Schulz, 2015), as physiological stress responses affect the activity of peripheral organs, and metabolic and immune functions via neural and endocrine pathways.

It is important to note that ascending and descending information between the brain and the body is continuously exchanged, and not only during interoception and stress. Homeostatic regulation of peripheral physiological processes via neural and endocrine feedback loops typically work without the involvement of higher CNS functions, unless they are working beyond a ‘normal’ range of functioning. If they exceed a certain threshold, they may be pushed into awareness of the organism. A physiological stress response elicits the activation of the SAM and HPA axes, which is far above the ‘normal’ range of homeostatic regulation, a process that has been named ‘allostasis’ (McEwen, 2004). Stress can, therefore, be conceptualized as descending information on the brain–body axis of increased amplitude as compared to a state of homeostasis, which may shift into focus of awareness. Interoception implicates that awareness is focused on bodily functions, even if they work in a normal, homeostatic range.

In summary, the relationship between interoception and stress involves a bi-directional communication on the brain–body axes. The aims of this review are to elucidate (1) the bi-directional communication between the brain and the body and (2) its role in the etiology of mental disorders. Using a systematic literature review we investigated the relationship between descending (stress) and ascending (interoception) transmission on the brain–body axis. We further address the question whether the dysregulation of bi-directional communication on the brain–body axis facilitates the generation of physical sensations. Furthermore, we defined psycholological processes (anxiety, attention, learning, decision making) that have previously been shown to be associated with stress.

## Method

A systematic literature review was performed on the relationship between interoception and stress, and between interoception and mental disorders, which are associated with stress and physical symptoms (i.e., depression, PD, SDs, dissociative disorders) using Pubmed/Medline, PsycInfo and PSYNDEX. Primary keywords used were “interoception,” “heartbeat perception/detection,” “visceral perception” and “symptom perception.” To address research objective (1), primary keywords were combined with secondary keywords “stress,” “anxiety,” “autonomic/sympathetic nervous system,” “nor/epinephrine,” “HPA axis” and “cortisol.” To address objective (2), primary keywords were combined with keywords “depression/depressive,” “panic/agoraphobia,” “somatoform,” “fucntional disorder/syndrome/complaint,” “medically unexplained symptoms,” “dissociation/dissociative” and “depersonalization/derealization.” On abstract-based search, studies were identified to contain at least one interoceptive indicator and either an experimental task that is considered inducing stress in a laboratory environment, and/or a player in physiological stress axes, and/or self-reported stress, and/or the inclusion of at least one of the respective mental disorders. Secondary literature as provided by the identified papers was also screened according to these criteria. For supplementary sections addressing the relationship between interoception psychological processes, which are affected by stress (anxiety, attention, learning, decision making), non-exhaustive literature searches were performed. Therefore, primary keywords were combined with secondary keywords dependent on the topic of the respective section. Based on the identified literature, interoceptive research methods are summarized and discussed in Section “Terminology and Definition,” which precedes the integration of the main results. Extraction of literature was performed independently by both authors.

## Results

The search on the relationship between interoception and stress resulted in 24 studies, which are exhaustively reported below (see **Table 1**). Furthermore, we identified nine studies on interoception in depression (one review), 14 studies on interoception in PD (three reviews), 12 studies on interoception and somatoform/functional disorders, and five studies on interoception in depersonalization disorder (DPD; one hypothesis paper). Due to space limitations the search was restricted to studies investigating the relationship between interoception and stress or stress-related processes.

**Table 1:** Summary on empirical research papers addressing acute or chronic stress and interoception.

Reference	Stress intervention	Interoceptive indicator	Study type	Sample size	Main findings on stress and interoception
Durlik et al. (2014)	Anticipation of public speakting (10 min)	Schandry-based heartbeat perception task	Control-/stress group (between design)	62 (42 f)	Increase of IA during anticipation
Eichler and Katkin (1994)	Mental arithmetic task (1 min; 3 min rest)	Whitehead-/Katkin-based heartbeat perception tasks	Baseline-/stress period (within factor); fixed order; good vs. poor heartbeat perceivers (quasi-experimental factor)	48 m; 23 good vs. 25 poor perceivers	Good perceivers show higher PEP and HI, and marginally higher CO stress response
Elsenbruch et al. (2010a)	Public speaking paradigm (5 min prep., 5 min speaking), fMRI compatible	BOLD response to painful and non-painful rectal stimulation	Stress-/relaxation period (within design)	15 (f) IBS patients, 12 (f) healthy controls	During stress increased activation of insula, midcingulate cortex, and ventrolateral prefrontal cortex in IBS
Fairclough and Goodwin (2007)	4 × mental arithmetic task (3 min each)	Whitehead-based heartbeat perception task	Stress-/relaxation session (within design); counterbalanced order	40 (20 f)	Decrease of IA after stress in females
Gupta et al. (2014)	No intervention; early life stress assessed via Early Trauma Inventory	Functional connectivity in six resting state networks (BOLD)	IBS/healthy control group (between design); correlative design (early life stress and brain network activity)	58 (28 f) IBS patients, 110 (72 f) healthy controls	Correlation between early life stress and activation of salience/executive control network in IBS patients
Gray et al. (2007)	Mental arithmetic task	Heartbeat-evoked potentials (arithmetic/control task)	Baseline-/stress period (within design)	10 m with cardiac dysfunction	Change of cardiac output correlated with HEP changes during stress; no effect of stress on HEPs
Herbert et al. (2010)	Mental arithmetic task (first 5 min)	Schandry-based heartbeat perception task	Baseline-/stress period (within factor); fixed order; good vs. poor heartbeat perceivers (quasi-experimental factor)	38 (19 f); 19 good vs. 19 poor perceivers	Stress-induced increase of HR, PEP and HI correlated to IA; good perceivers show higher HR and PEP stress response
Jones and Hollandsworth (1981)	Physical exercise (bicycle; to achieve 75% increase in heart rate)	Identification of correct or false heart rate feedback	Baseline-/exercise period (within factor); exercise: tennis players, distance runners, control (quasi-experimental)	36 (18 f)	Distance runners had highest IA during baseline; IA in tennis and control group after exercise
Kindermann and Werner (2014)	3 × 3 min mental arithmetic test (PASAT)	Schandry-based heartbeat perception task	Good vs. poor heartbeat perceivers (quasi-experimental factor)	20 good vs. 20 poor perceivers	Good perceivers show higher negative affect during stress; no difference in heart rate response
Moor et al. (2005)	Epinephrine; esmolol; norepinephrine; sodium-nitroprusside (dose-response)	Whitehead-based heartbeat perception task	Placebo-controlled study (within design); fixed order	24 m	Nitroprussid and epinephrine increased, esmolol decreased IA
Pohl et al. (1998)	Preparation for public speech	Report of hypoglycemia symptoms after insulin bolus	Placebo-controlled study (2 × 2 between design: insulin vs. placebo; stress vs. control intervention)	40 m	Less accurate detection of insulin adminstration and recudes hypoglycemia symptoms after stress
Pollatos et al. (2007b)	Isometric handgrip exercise	Schandry-based heartbeat perception task	Baseline-/exercise period (within factor); high vs. low cardiovascular responders (quasi-experimental factor)	18 m; 9 high vs. 9 low cardiovascular responders	Higher IA in high than low responders; IA correlated with response in HR, SBP, CO and PEP
Richards et al. (1996)	Physical exercise (stepping machine; 1 min)	Correlation between actual and perceived changes in heart rate	Relaxation-/exercise condition (within); panic vs. control individuals (group); intra-correlation design	26 panic patients (14 f); 14 healthy controls (9 f)	Higher IA (intra-correlation) after exercise, no interaction with group factor
Rosenberger et al. (2009)	Public speaking paradigm, fMRI compatible	BOLD response to painful and non-painful rectal stimulation	baseline-/stress period (within design); randomized order	14 f	Stress induces differences in activity of right posterior cingulate and S1, and left thamalus during painful stimulations
Schandry and Specht (1981)	Public speaking test	Schandry-based heartbeat perception task	Baseline-/stress period (within design)	41	Increase of IA after stress
Schandry et al. (1993)	0–90° tilt; ergometric bicycle exercise (0, 25, 50, 75 W)	Schandry-based heartbeat perception task	0, 25, 50, 75 W; fixed order conditions (within design)	25 (14 f)	IA correlated with HR, SV, HI and momentum over all conditions
Schmitz et al. (2012)	Public speaking test (3 min)	Schandry-based heartbeat perception task	Low vs. high social anxiety (quasi-experimental factor)	40 (21 f) children; 20 high vs. 20 low socially anxious	After stress high socially anxious show higher IA than low socially anxious children
Schulz et al. (2011)	3-min socially-evaluated cold pressor test (0–3°C)	Cardiac modulation of startle	Control-/stress group (between design)	38 (24 f)	Earlier CMS effect after stress
Schulz et al. (2013a)	3-min socially-evaluated cold pressor test (0–3°C)	Schandry-/Whitehead-based heartbeat perception task	Control-/stress group (between design)	42 (29 f)	Higher Schandry-, lower Whitehead-based IA after stress
Schulz et al. (2013b)	4 mg of intraveneous cortisol	Heartbeat-evoked potentials (rest)	Placebo-controlled study (within design), counterbalanced order	16 m	Higher HEPs after cortisol in open than in closed eyes
Shao et al. (2011)	10-min cold pressor test (10°C)	Heartbeat-evoked potentials (rest/control task/CP)	Baseline-/stress period (within design); randomized order	21 (9 f)	Decrease of HEPs during CP
Steptoe and Vogele (1992)	5-min mental arithmetic, 3-min cold pressor test	Correlation between actual and perceived physiology	Baseline-/arithmetic/cold pressor condition (intra-correlation design)	30 f	No effect of stress on IA reported; IA correlated with information-seeking coping style
Stevens et al. (2011)	Anticipation of public speaking	Schandry-based heartbeat perception task	Resting-/anticipation period (within factor); low vs. high social anxiety (quasi-experimental factor)	48 (25 f); 24 high vs. 24 low socially anxious	Marginal increase of IA after stress; no interaction between stress and anxiety groups
Sturges and Goetsch (1996)	7-min mental arithmetic task	Schandry-based heartbeat perception task	Baseline-/stress period (within factor); low vs. high anxiety sensitivity (between factor)	59 f; 29 high vs. 30 low anxiety sensitive	Higher IA after stress in high than in low anxiety sensitivity

## Terminology and Definition

Sensory information from the body can originate from different sources, i.e., (1) from exteroceptors, e.g., located in the skin, which transmit somatosensory information, (2) from proprioceptors, e.g., receptors in the spindles of skeletal muscles, and (3) from interoceptors, e.g., mechano-, chemo-, thermo-, or metabo-receptors within visceral organs (Schulz, 2015). In fact, signals from interoceptive and somatosensory sources are likely to be integrated in CNS structures to construct a representation of bodily processes (Craig, 2002, 2009; Wiens, 2005). This could explain, for instance, why patients with damaged or degenerated afferent autonomic nerves show impaired perception of visceral sensations (Pauli et al., 1991; Leopold and Schandry, 2001; Schulz et al., 2009a), while largely maintaining their affective experience (Cobos et al., 2004; Heims et al., 2004), which is assumed to be associated with interoception. However, the current review makes the pre-assumption that visceral-afferent signal transmission plays an important, yet not an exclusive role in the central representation of bodily signals.

There has been a debate in the literature on the taxonomy of facets of interoception. Garfinkel and Critchley (2013) have suggested separating the subjective tendency to be focused on interoceptive sensations from the actual accuracy in interoceptive tasks. They call the former ‘interoceptive sensibility’ and the latter ‘interoceptive sensitivity.’ In between, a third level was proposed, which was hypothesized to represent the degree of predictive value between interoceptive sensibility and sensitivity, which was named ‘interoceptive awareness.’ This taxonomy is in partial disagreement with earlier studies, which consider accuracy in heartbeat perception tasks to be an index of ‘interoceptive awareness.’ It is undisputed that awareness is required to perform in heartbeat perception tasks, but empirical evidence clearly shows that ‘awareness’ to interoceptive sensations and performance in heartbeat perception are partially unrelated (Khalsa et al., 2008; Ceunen et al., 2013). Although the differentiation between ‘sensibility’ and ‘sensitivity’ is plausible, in our opinion the measure indexed by interoceptive tasks should be called ‘interoceptive accuracy’ (IA) instead. ‘Interoceptive sensitivity’ originates from signal detection theory and implies the minimum threshold to detect an interoceptive signal from background noise (Farb et al., 2015). In contrast, ‘IA’ refers to the objective performance in counting interoceptive sensations or in discriminating and interoceptive from an exteroceptive sensation (see below; Ceunen et al., 2013). ‘IA’ does not suggest that a specific score reflects ‘sensitivity’ or ‘awareness,’ as some individuals may be hyper-sensitive or show extreme awareness to interoceptive sensations, which leads to misinterpretation of signals and thus to lower accuracy, while others may rarely focus awareness toward interoceptive stimuli, but may be highly accurate in performing heartbeat perception tasks (Schulz, 2015). In a revised version of the theory, performance in heartbeat perception tasks was now called ‘IA’ (Garfinkel et al., 2015), which is also reflected in this review.

The perception of bodily processes involves at least three consecutive stages: (1) afferent neural signals from the body, such as visceral organs, (2) the direction of attention toward bodily sensations, and (3) the evaluation of these signals and their integration into psychological processes. As suggested by Vaitl (1996) these three stages can be further differentiated. For instance, visceral-afferent signals involve the stimulation of interoceptors at the peripheral organ, the neural transmission of these signals from interoceptors to CNS structures, and finally the CNS representation of these signals. The evaluation of bodily sensations and their integration into psychological processes are associated with subjective reports of physical sensations, and an individual learning history concerning these sensations.

## Methodology to Assess Interoception

### Interoceptive Sensibility

The meta-cognitive tendency (i.e., interoceptive sensibility) to focus on interoceptive sensations is commonly assessed via questionnaires. The majority of existing questionnaires is designed to assess physical symptoms and clinically altered interoception in mental disorders, such as eating disorders (Eating Disorder Inventory-2: Garner, 1991) or SDs (Somatosensory Amplification Scale: Barsky et al., 1990). Instruments for the assessment of ‘normal’ interoception are scarce. Several existing studies have focused on the Body Perception Questionnaire by Porges (1993). However, there are no sufficient estimations for psychometric properties available for this questionnaire. The recently developed Multidimensional Assessment of Interoceptive Awareness (MAIA; Mehling et al., 2012) incorporates a number of interoceptive aspects (noticing, non-distracting, not-worrying, attention regulation, emotional awareness, self-regulation, body-listening, trusting) and is thus designed to study ‘normal’ and clinically dysregulated interoception.

### Interoceptive Accuracy

The majority of empirical data on interoception is based on ‘IA’ as assessed by experimental paradigms that require subjective reports of physical sensations. Regarding the cardiovascular system, there are two main categories of tasks: (1) heartbeat counting tasks and (2) heartbeat discrimination tasks, as summarized elsewhere (Schulz, 2015). Heartbeat counting tasks were developed by Schandry (1981), who named the task ‘mental tracking test.’ The task consists of time intervals of different duration (original version: 35, 45, and 55 s), during which participants are instructed to silently count the number of their heartbeats. This number is later compared to the actual number of heartbeats in this interval. The absolute value of the difference between both is divided by the actual number of heartbeats and this ‘inaccuracy’ index is then subtracted from 1, which results in the accuracy score of the heartbeat counting task. It has been repeatedly demonstrated that the accuracy score depends on the wording of the instruction given. The comparison between a standard instruction (“count all heartbeats you feel in the body”) and a strict instruction (“count only those heartbeats about which you are sure of”) suggests that an individual’s knowledge on their heart rate and capacity to accurately estimate the duration of time intervals may be important factors for the accuracy in heartbeat counting tasks (Ehlers et al., 1995; Ehlers and Breuer, 1996). Heartbeat discrimination tasks were originally developed by Brener and Jones (1974) and further elaborated by others (Whitehead et al., 1977; Katkin et al., 1981; Brener and Kluvitse, 1988). In these tasks participants are asked to judge whether a set of consecutive exteroceptive stimuli (e.g., lights, tones, tactile stimuli) appear simultaneously with their own heartbeats (S+ trials) or not (S- trials). The available variants of this task mainly differ in the setup of S- trials, since some present exteroceptive stimuli with a fixed delay to heartbeats, while others simulate a set of artificial stimuli without any relation to the actual heartbeats. Previous research has yielded mixed findings regarding the question whether IA assessed by both tasks families are correlated: some report a moderate positive correlation (Knoll and Hodapp, 1992; Schaefer et al., 2012; Hart et al., 2013), while others did not find any association (Phillips et al., 1999; Schulz et al., 2013a; Michal et al., 2014).

Beyond heartbeat perception tasks, subjective reports are also required for paradigms incorporating the perception of respiratory resistances (Pappens et al., 2013; Tsai et al., 2013; Petersen et al., 2014), of gastric distensions (Rosenberger et al., 2009; Elsenbruch et al., 2010b), and of non-specific skin conductance fluctuations (Andor et al., 2008). In our view, the outcomes of these tasks reflect all stages of interoception at any given point in time (i.e., learning, report, awareness, CNS representation, visceral-afferent signal transmission, and stimulation of interoceptors). We would argue, therefore, that in addition to these measures, methods are required that enable the differentiation of interoception stages, e.g., between those at lower and higher levels of awareness.

### Psychophysiological Methods

To date, at least three psychophysiological indicators are available to assess interoceptive signal transmission below the threshold of consciousness: (1) Baroreflex-sensitivity (BRS) quantifies the changes in heart period in responses to changes in arterial blood pressure (Robbe et al., 1987). As this brainstem reflex requires intact baro-afferent signals transmission, BRS is considered an indicator of the integrity of afferent autonomic nerves (Frattola et al., 1997). The disadvantage of this indicator is the fact that it reflects both the afferent and efferent branch of the baroreflex at a time. (2) Using the ‘cardiac modulation of startle’ (CMS) paradigm we could show that visceral-afferent neural signals from the cardiovascular system may affect acoustic startle responses (Schulz et al., 2009a,b,c, 2011). This modulation is largely diminished in individuals with degeneration of afferent autonomic nerves (Schulz et al., 2009a) and may also involve afferent signals from other organ systems (Schachinger et al., 2009; Schulz et al., 2013b, 2015c). The CMS effect can be interpreted as an indicator of visceral-afferent neural signal processing at brainstem level, as it probably only involves brainstem mechanisms and occurs below the threshold of consciousness, (3) viscerally evoked brain potentials are considered indicators for the cortical representation of afferent signals from visceral organs. They can be further differentiated into heartbeat-evoked potentials (HEPs), which are related to the processing of heartbeats (Schandry et al., 1986; Leopold and Schandry, 2001), and respiratory-related potentials that are observed when an inspiratory or expiratory occlusion is presented (Davenport et al., 1986; Chan and Davenport, 2010; von Leupoldt et al., 2010).

Another psychophysiological indicator of interoception is the single trial covariance between event-related brain potentials (N300) and changes in heart period (∼3–4 s after stimulus) in response to feedback in decision making tasks (Mueller et al., 2010a, 2012, 2013). This indicator is limited, however, to the use with decision making paradigms involving feedback. The theoretical background for this effect is provided by Damasio’s (1994) “somatic marker hypothesis” positing that afferent information from visceral organs is integrated in the evaluation of alternatives in decision making. It is plausible that the covariation between cortical and cardiac responses is specifically relevant for decision making paradigms tasks, although it is for future research to show whether this effect also extends to other embodied cognitions.

## Synthesis of Findings on Interoception and Stress

**FIGURE 1:**
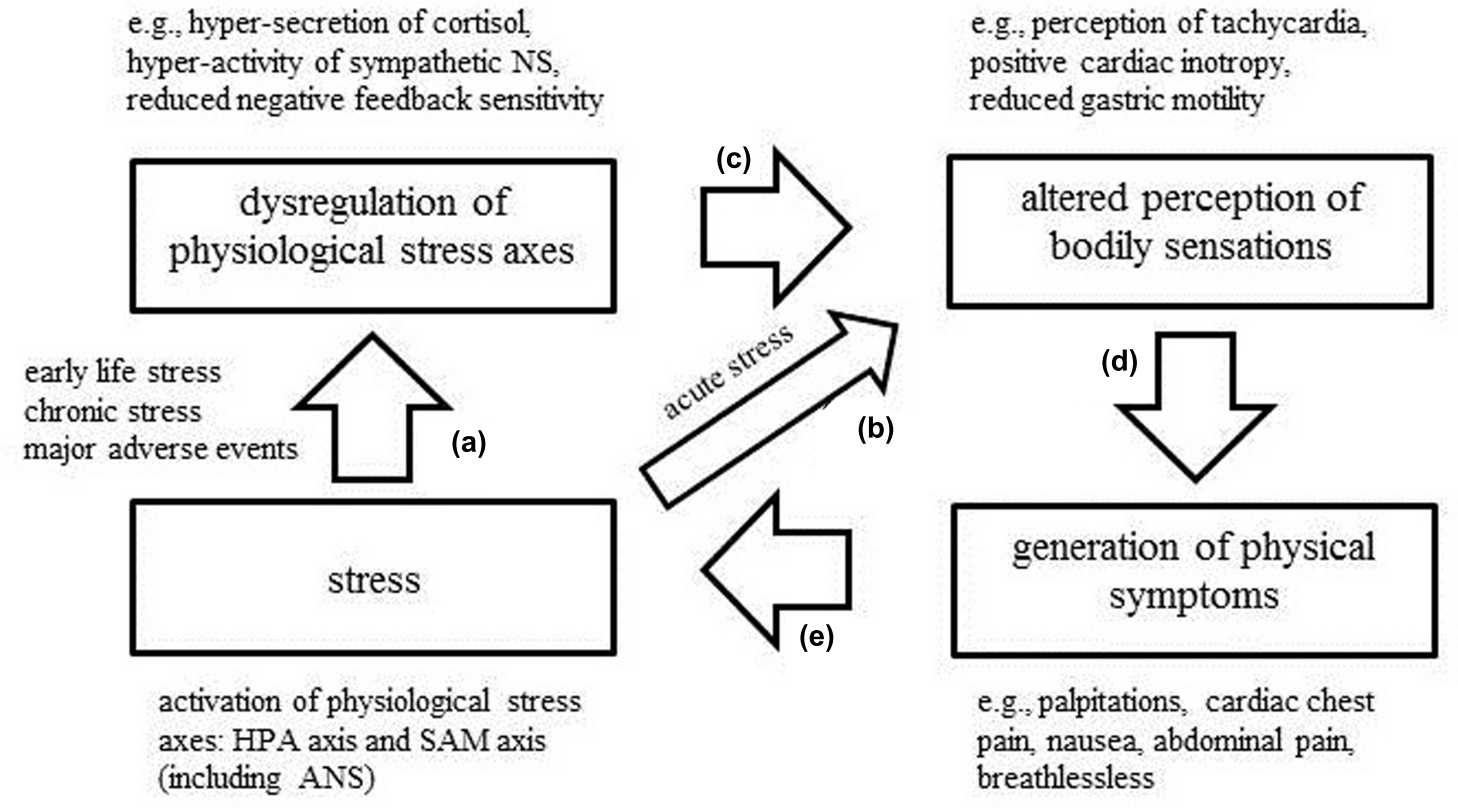
**Synthesis of findings on acute and chronic stress, dysregulation of physiological stress axes, altered interoception and the generation of physical symptoms into model comprising a positive feedback loop**.

Based on the findings from the current review, we propose a conceptual framework that allows for the explanation of the role of the dysregulated association between interoception and stress for the generation of physical symptoms (see **Figure 1**). In this model we postulate that stress is the initial point of a positive feedback cascade. In case of an acute stressor, the cascade will follow the pathway b–d–e. In particular, acute stress will activate physiological stress axes, such as the release of cortisol and activation of peripheral organs by sympathetic mechanisms. The altered stimulation of peripheral interoceptors and central effects of cortisol and noradrenergic structures will affect the perception of bodily sensations (e.g., perception of tachycardia or positive cardiac inotropy). Alterations in the perception of bodily sensations may temporarily feed into the experience of physical symptom during acute stress, for instance, palpitations, nausea or breathlessness. The perception of these symptoms could then be perceived as stressful and thus contribute to the maintenance of a stress response. However, due to the limited duration of an acute stress response, the cascade will be disrupted after the stress-eliciting stimulus has disappeared. In case of the confrontation with a chronic, early or major life stressor, the dysregulation of physiological stress axes (e.g., chronic hyper-activation of the HPA axis or the SAM axis) is implied and the model will follow the pathway a-c-d-e. Once the manifestation of a stress axis disorder has occurred, the cascade cannot be easily disrupted anymore, even if the initial stressor has disappeared. In detail, if the organism experiences chronic dysregulation of a stress axis, this state may permanently induce altered perception of bodily sensations and contribute to the manifestation of physical symptoms, whose perception consolidates the experience of stress. This model has an intended similarity to the model of somatosensory amplification by Barsky et al. (1988) and Barsky (1992), which emphasizes the importance of positive feedback mechanisms in activation, stress and physical symptoms. In contrast to the model by Barsky et al. (1988) however, the current model makes no assumptions about the role of attention to bodily sensations, although the latter could be conceived of as one of several psychobiological mechanisms. The core assumption in the current model concerns the interrelation of ascending and descending signals on the brain body axis in a positive feedback fashion. The following paragraphs will summarize empirical findings supporting this model.

### Chronic Stress and Dysregulation of Physiological Stress Axes (Path *a*)

It is well documented in the literature that prolonged exposure to psychosocial stress, early life adversity and/or major adverse events represent major risk factors for the dysregulation of the physiological stress axes. The regulation of the HPA axis can be differentiated into three different mechanisms (Li-Tempel et al., submited): (1) baseline activity and circadian rhythmicity, such as morning or daily profiles in cortisol level, (2) reactivity of CRF, ACTH, and cortisol in response to acute stress, and (3) (negative) feedback sensitivity, such as the suppression of cortisol release by dexamethasone. In general, similar mechanisms also exist for the SAM axis. In particular, baseline concentrations of catecholamines can be assessed from blood or urine, indirectly from salivary samples via alpha-amylase, or psychophysiological indicators, such as pre-ejection period. The investigation of circadian rhythmicity is limited due to the very short plasma half-life of catecholamines (∼1–3 min.). Furthermore, the same indicators can be investigated in response to an acute stressor. Negative feedback mechanisms in the SAM axis are, for instance, central α2-adrenergic receptors. These receptors are critically involved in brainstem-relayed reflex circuits that regulate the homeostasis of sympathetic and parasympathetic output, e.g., the arterial baroreflex (Sved et al., 1992). As the players involved in SAM axis regulation involve complex neural and endocrine pathways and are, therefore, more heterogeneous, the same is true for their indicators. For example, some parameters, such as low frequency heart rate and blood pressure variability are more sensitive to central sympathetic activation, while others, such as pre-ejection period are indicative of peripheral sympathetic activation (e.g., circulating catecholamines; Schachinger et al., 2001).

Early life and chronic stress, as well as major adverse life events, are consequently associated with dysregulation in all three types of mechanisms and in both stress axes (McEwen, 2000). Regarding HPA axis baseline and circadian rhythmicity, for instance, chronic stress, resulting from caregiving to family members, is reflected in altered cortisol awakening response (CAR; de Vugt et al., 2005), reduced cortisol release during daytime (Bella et al., 2011), and elevated hair cortisol levels (Stalder et al., 2014). In chronic stress associated with peer bullying and victimization reduced CAR was observed (Knack et al., 2011). Aberrant cortisol responses to acute stressors have been also found in family caregivers (De Andres-Garcia et al., 2012) and those exposed to peer victimization (Knack et al., 2011). Feedback sensitivity by dexamethasone suppression is altered in individuals exposed to high work stress, as indicated by self-reported burnout symptomatology (Pruessner et al., 1999), and after major traumatic experiences (Yehuda et al., 1993). Regarding the SAM axis, chronic stress by caregiving is associated with baseline indices of peripheral sympathetic activation (Cacioppo et al., 2000). Work stress as indicated by over-commitment to work is related to lower baseline norepinephrine levels (Wirtz et al., 2008), but increased activity in psychophysiological indicators of peripheral epinephrine circulation (Vrijkotte et al., 2004) and lower central parasympathetic output (Chandola et al., 2010). Furthermore, lower norepinephrine responses to acute stress have been found in individuals high in over-commitment, suggesting high work-related stress (Wirtz et al., 2008). Individuals exposed to early life stress show blunted reactivity to acute stress in indicators reflecting peripheral sympathetic activation (i.e., pre-ejection period; McLaughlin et al., 2014). Finally, there is evidence from animal models of reductions in the expression of α2-adrenergic receptors (which are involved in SNS-down-regulation) in response to chronic psychosocial stress (Flugge et al., 2003). The arterial baroreflex circuit, which is partially mediated by α2-adrenoceptors (Sved et al., 1992), shows reduced functioning under conditions of chronic work stress (Thomas et al., 2004).

Chronic stress and the associated dysregulation of physiological stress responses represent important factors for the development and maintenance of mental disorders, in particular those, which are associated with symptoms of altered perception of bodily sensations (e.g., depression, PD, SDs, dissociative disorders). Nevertheless, the current literature lacks a discussion of the mechanisms underlying the relationship between stress, interoception and physical symptoms. For example, it has been repeatedly suggested that somatic syndromes, e.g., chronic fatigue syndrome (CFS) or fibromyalgia (FMS), are associated with lower cortisol responsiveness or increased feedback sensitivity (Cleare, 2004; Tanriverdi et al., 2007; Wingenfeld et al., 2008; Tak et al., 2011), but only few hypotheses addressing the mechanisms of how cortisol may contribute to the generation of physical symptoms. One such hypothesis addresses immunological changes that are associated with dysregulation of the HPA axis (Fries et al., 2005). It is possible that the modulation of pro- and anti-inflammatory cytokines may contribute to somatic syndromes, such as FMS. Another explanation focuses on the importance of hypocortisolism in CFS and argues that cortisol mobilizes energy resources to overcome daily demands, which are insufficiently available in CFS (Tak and Rosmalen, 2010). However, both explanations are focused on peripheral bodily processes and do not incorporate possible effects of stress on cortical representation and perception of bodily sensations. The possibility that cortisol could also modulate the perception of bodily sensations was first raised by Rief et al. (1998b).

### Acute Stress and Interoception (Path *b*)

#### Laboratory Stress Tests

A broad variety of methods has been used to induce acute stress in laboratory settings (Steptoe and Vogele, 1991). Mental arithmetic tasks elicit ‘psychological’ stress generally by inducing mental load and specifically by involving central control mechanisms over autonomic processes (Moriguchi et al., 1992; Sloan et al., 1995). Psychosocial stressors that emphasize public speaking challenges, such as the Trier Social Stress Test (TSST), are known to provoke an intense response of the HPA axis (indexed e.g., by an increase of salivary cortisol; (Kirschbaum et al., 1993; Kudielka et al., 2004) and activation of the cardiovascular system by sympathetic mechanisms. Given the TSST’s comparatively long duration of ∼15 min on the one hand, and the very fast acting release and effects of catecholamines (i.e., seconds) on the other hand, specific sympathetic processes involved in the TSST are difficult to disentangle. As the cold pressor test induces stress associated with the experience of ischemic pain, these results in specific cardiovascular response patterns, which can partially be attributed to the experience of pain, but also to the vaso-constricting effect of cold water (Streff et al., 2010). The socially-evaluated cold pressor task (SECPT) attempts to integrate psychosocial and physical stressors, and has been demonstrated to elicit significant increases in saliva cortisol (Schwabe et al., 2008; Lass-Hennemann et al., 2010, 2011; Larra et al., 2014), as well as sympathetic activation, as indicated by an increase of heart rate, and systolic and diastolic blood pressure (Schwabe et al., 2008; Schulz et al., 2011, 2013a) and a decrease of pulse-transit time and BRS (Schulz et al., 2011). To understand possibly diverging results on the impact of acute stressors on indicators of interoceptive signal processing and IA, differences in the methods used to induce stress and to assess interoception have to be taken into account (Steptoe and Vogele, 1992).

#### Interoceptive Accuracy Assessed with Heartbeat Perception Tasks

Interoceptive accuracy as estimated by Schandry-based heartbeat tracking tasks typically increase under acute stress. While this effect has been observed in anticipation of (Durlik et al., 2014) and after a public speaking test (Schandry and Specht, 1981), one study only found a marginal increase in IA during anticipation (Stevens et al., 2011). The importance of the amplitude of cardiovascular responsiveness during stress for the enhancement of IA is underlined by the positive correlation between IA and responsiveness in heart rate, pre-ejection period and the Heather index in a mental arithmetic task (Herbert et al., 2010). A similar association between cardiovascular responsiveness and IA could also be shown for the heartbeat discrimination task (Eichler and Katkin, 1994). Acute responses to stress include sympathetic effects on the cardiovascular system, and a delayed increase in cortisol secretion, if a psychosocial challenge (e.g., public speaking) is involved. There are also attentional and affective changes in response to stress (see Psychobiological Mechanisms Involved in Interoception and Stress), which may interact with interoception. For instance, individuals with high IA in heartbeat perception show higher negative affect during stress than individuals with low IA (Kindermann and Werner, 2014). Furthermore, it has been shown that IA increases after physical exercise (Jones and Hollandsworth, 1981; Richards et al., 1996), and that the same correlation between cardiovascular reactivity measures (cardiac output, heart rate, systolic blood pressure, Heather index, momentum, stroke volume) to exercise and IA exists (Schandry et al., 1993; Pollatos et al., 2007b). It could be argued, therefore, that peripheral sympathetic activation during stress represents the core factor of this relationship. In contrast to these results using Schandry-based heartbeat tracking tasks, IA as assessed with heartbeat discrimination tasks, is reduced in women performing a mental arithmetic task (Fairclough and Goodwin, 2007). Using the SECPT we could replicate these seemingly contradictory findings, i.e., an increase in IA after stress as assessed by the Schandry-heart beat tracking task and a decrease in IA when using a visual heartbeat discrimination task (Schulz et al., 2013a). As the results obtained in these studies seems to be IA-paradigm dependent the observed differences are most likely associated with task specific characteristics: while heartbeat tracking tasks require participants to only focus their attention on visceral sensations, the heartbeat discrimination task involves the concurrent monitoring of visceral sensations and external signals. According to the competition-of-cues model by Pennebaker and Lightner (1980), interoceptive and exteroceptive signals compete for a limited resource, i.e., attention. As acute stress may narrow attentional resources and favor attention to task-relevant stimuli (Chajut and Algom, 2003; Plessow et al., 2011), the multisensory integration of information as required by the heartbeat discrimination task may be impaired by stress exposure. This assumption is further supported by the observation that after the preparation for public speech, individuals exhibited reduced accuracy in detecting an insulin-induced hypoglycemic state (Pohl et al., 1998), which also requires attention focused on multiple symptoms. In summary, there is evidence for two mechanisms relevant for IA in response to acute stress: (1) peripheral sympathetic activation, which induces an increased stimulation of cardiac interoceptors (e.g., arterial baroreceptors) and intensifies afferent signal transmission from the cardiovascular system, and (2) attention focus on visceral sensations, which may improve the detection of visceral signals, but diminish the integration of signals from other sensory modalities. When both effects are opposed, as e.g., in the heartbeat discrimination task, it appears that attention represents the more important determinant of IA. This dissociation emphasizes the need for the separate assessment of visceral-afferent signal transmission and representation on the one hand, and attention to visceral signals on the other hand.

#### Psychophysiological Indicators of Interoceptive Signal Processing

While HEPs and CMS may serve as indicators of visceral-afferent signal transmission independent of attention to these signals, empirical data on stress effects on these indicators is scarce. In one study we investigated the impact of the SECPT on CMS (Schulz et al., 2011). After stress exposure we observed CMS to occur earlier within the cardiac cycle (0–200 ms instead of 200–300 ms after the R-wave); yet, contrary to our expectations, there was no *amplification* of the CMS effect. Nevertheless, as the CMS reflects the intact transmission of afferent neural signals from the cardiovascular system at brainstem level (Schulz et al., 2009a), these findings suggest that the *amplitude* of representation of afferent signals may be unchanged by stress. Similar findings have also been reported for HEPs, with reduced amplitudes during a long-term, but mild cold pressor test (10-min, 10°C) and a return to baseline levels after the cold pressor test (Shao et al., 2011). This response pattern may be explained by participants focusing their attention during the cold pressor primarily on the pain experience, and thus away from cardiovascular sensations. Nevertheless, after termination of stress exposure, the expected sympathetic response did not affect HEP amplitudes. Gray et al. (2007) investigated the impact of a mental arithmetic task on HEPs in 12 men with cardiac dysfunction, and found a positive relationship between changes in cardiac output and HEPs, but no effect of the stress task on HEP amplitudes. To the best of our knowledge, there is only one study reporting an effect of acute stress on a psychophysiological indicator of interoception: after an MRT-compatible public speaking test, the BOLD response to painful rectal distensions was different in the right posterior cingulate and right somatosensory area, as well as the left thalamus (Rosenberger et al., 2009). Despite the large methodological differences across these studies in terms of laboratory stress tasks employed, organ systems investigated and derivation of interoceptive indicators, it may be speculated that acute stress specifically changes the processing of visceral-afferent signals from the gastrointestinal system. As the majority of studies demonstrate no *quantitative* change of raw representations of visceral-afferent signals by stress, it is still plausible that stress induces a *qualitative* change in interoception, as suggested by our findings (Schulz et al., 2011). It can be summarized that, contrary to expectations, current results implicate that there is no main effect of acute laboratory stress on the *amplitude* of psychophysiological indicators of interoception after the stressor. Possible reasons for the null findings in these studies could involve the limited effectiveness of mental arithmetic and 10°C cold pressor tasks, as well as the limited time frame after stress during which responses were monitored. Given the heterogeneity of mechanisms induced by acute laboratory stress, it seems necessary to determine the differential role of the physiological stress axes on interoception. **Table 1** provides an overview of studies addressing the relationship of stress and indicators of interoception.

### The Sympatho-Adreno-Medullary Axis and Interoception

As described earlier, reactivity in pre-ejection period or Heather index to laboratory stress, (which may reflect peripheral sympathetic activation) is positively related to heartbeat perception (Eichler and Katkin, 1994; Herbert et al., 2010). However, as neither study derived heartbeat perception scores from a post-stress period, it can only be concluded that individuals with a general tendency for responding with peripheral sympathetic activation show higher IA in heartbeat perception tasks. To elucidate the contribution of both the central and peripheral sympathetic branch to heartbeat perception, the impact of different adrenergic agents on accuracy in a heartbeat discrimination task has been investigated by (Moor et al., 2005). In particular, sodium nitroprusside as α1-adrenergic antagonist, and norepinephrine as α1-agonist, as well as the β1-agonist epinephrine and the β1-antagonist esmolol were employed. Since β1-adrenoreceptors are located in the myocardium and are sensitive to circulating catecholamines, especially epinephrine, selective stimulation or de-stimulation represents a pharmacological model for peripheral sympathetic activity (Schachinger et al., 2001). Epinephrine increased and esmolol decreased IA as assessed by a heartbeat discrimination task as compared to placebo (Moor et al., 2005). This finding suggests that peripheral sympathetic activation enhances IA. Since epinephrine cannot cross the blood–brain barrier, two alternative ways of signal transmission are possible: first, β-adrenergic receptors localized at vagal nerve endings may directly be stimulated by circulating epinephrine (Mravec, 2011). Second, increased cardiac contractility may cause increased stimulation of cardiac interoceptors (e.g., baroreceptors), whose neural signals are transmitted over the nervus glossopharyngeus (Jänig, 2006). α1-adrenoreceptors are primarily located in the vascular musculature. Their stimulation induces an increase in vascular resistance and, therefore, in blood pressure. This information is relayed via the arterial baroreflex circuit in order to decrease heart rate accordingly for the maintenance of blood pressure level. The dis-stimulation of α1-adrenoreceptors causes the opposite effect. This pharmacological design is thus suitable to investigate the selective loading and unloading of arterial baroreceptors and the subsequent central sympathetic activation induced by this baroreceptor stimulation (Schachinger et al., 2001). Baroreceptor unloading by sodium nitroprusside resulted in an increase in IA as compared to placebo, while norepinephrine had no effect on heartbeat perception (Moor et al., 2005), which could be explained by increased central sympathetic output due to baroreceptor unloading. Central α2-adrenoreceptors, which are mainly located in the LC and NTS act as negative feedback mechanism in the central noradrenergic system (Rockhold and Caldwell, 1980). Hence, administration of a α2-adrenergic antagonist causes a concurrent activation of central and peripheral sympathetic mechanisms (Isaac, 1980; Philippsen et al., 2007). In a pilot study we found the α2-antagonist yohimbine to suppress the CMS effect (Schulz et al., 2007). This finding implies that concurrent central and peripheral sympathetic activation combined with increased arousal and vigilance as caused by noradrenergic activation may diminish the central processing of visceral-afferent signals at a low, presumably brainstem-associated level. Our own observations on α2-antagonism and IA suggest reduced IA after the administration of yohimbine. This observation may implicate that central noradrenergic activation, including increased alertness and vigilance, may also impair the cortical processing of visceral-afferent signals. Taken together, it can be concluded that there is a strong positive relationship between peripheral sympathetic activation and cardiac IA, but only a limited association between central sympathetic tone and IA. A strong activation of central noradrenergic mechanisms may even inhibit interoception, although concurrent peripheral activation would suggest a more intense stimulation of interoceptors.

### The HPA Axis and Interoception

Cortisol has been found to modulate HEP amplitudes within a timeframe of 1–17 min after infusion (Schulz et al., 2013c). More specifically, 4 mg of cortisol resulted in higher HEPs under open- compared with closed-eyes conditions, i.e., states of high vs. low alertness. This effect could eventually feed into a vicious circle of increased attention focus on physical symptoms, increased anxiety and higher levels of cortisol, and represent a psychobiological mechanism underlying positive feedback models of somatosensory amplification (Barsky et al., 1988). In this study HEPs were assessed during rest, without the conscious perception of heartbeats. The effect of cortisol administration on resting HEPs suggests that cortisol may affect the raw representation of visceral-afferent signals, independent of the conscious perception of heartbeats. Moreover, we did not observe any effect of cortisol on cardiovascular activation. This finding suggests that cortisol may selectively modulate the central, presumably cortical, representation of visceral-afferent signals, while the peripheral origination of these signals remain unaffected. Interestingly, in a complementary study no effect of 1.5 mg cortisol on the CMS was observed within the same time frame (Schulz et al., 2010). Since the CMS is assumed to reflect visceral-afferent signal transmission at brainstem level, one could speculate that the effects of cortisol on visceral-afferent signal relaying are restricted to the cortex. However, it needs to be acknowledged that the dosages of administered cortisol were not identical. Furthermore, the first study covered a time frame of up to 37 min and therefore includes possible genomic effects, while the second study focused on non-genomic effects only. The importance of cortisol for the cortical representation of visceral-afferent signals is further emphasized by the negative relationship between basal cortisol level and HEP amplitudes of *r* = -0.29 (Schulz et al., 2015b). Despite the fact that this result was based on a mixed sample of healthy individuals and patients with DPD, the correlation was unaffected by the diagnosis, or depression and anxiety scores. It can be summarized that acute cortisol administration tends to increase HEP amplitudes (when eyes are open), presumably via a non-genomic mechanism, while basal cortisol level shows a negative relationship with HEPs. One may speculate that the effect of cortisol on the cortical representation of visceral-afferent signals reverses into long-term cortisol elevations. Interestingly, these opposite effects contrast with findings in pain research: in experimental short-term manipulation of cortisol, an oral administration of 40 mg of cortisol reduces pain sensitivity (Michaux et al., 2012), while cortisol blockade by metyrapone intensifies pain perception (Kuehl et al., 2010). Meanwhile, in chronic pain syndromes, such as FMS, also reduced cortisol baseline levels and hyper-suppression by dexamethasone were observed (Wingenfeld et al., 2007, 2008). This possible dissociation in cortisol relationships between ‘normal’ visceral-afferent neural transmission and pain has to be acknowledged, since interoception and pain processing only partially share neural structures (Craig, 2002). Future research should clarify whether acute cortisol release may selectively favor the representation of ‘normal’ visceral-afferent transmission, which may reverse in long-term increases of cortisol levels. The majority of the existing literature on processes of body, symptom and pain perception concentrates on the role of cortisol, the final product of HPA axis activation. However, it needs to be taken into account that the role of CRF and ACTH on interoceptive processes remains unclear and possible differences in acute and chronic cortisol levels may also be attributed to feedback-induced changes in CRF or ACTH.

Taken together, there is considerable evidence to show that acute stress and players of physiological stress axes may alter the perception of bodily sensations. Based on our model, we propose that acute stress may induce an acute alteration of physical sensations and transient symptoms specific to the stress, which constitute the subjective experience of the stress response (e.g., tachycardia, palpitations, nausea, etc.). However, these symptoms disappear when the stress is of limited duration.

### Dysregulation of Physiological Stress Axes and Interoception (Path *c*)

So far there is only partial and indirect evidence for a direct effect of dysregulated stress axes on altered perception of bodily sensations. Earlier findings suggest that chronic stress in healthy individuals is not related to heartbeat perception accuracy (Schulz et al., 2013a) or sensitivity for gastric stimulations (Rosenberger et al., 2009). The direct pathway between chronic stress and altered perception of bodily sensations was, therefore, omitted in our model. It should be noted, however, that moderately elevated levels of self-reported chronic stress in healthy individuals, as reported in the former studies, are unlikely to be accompanied by a dysregulation of the physiological stress axes.

Our assumption that the dysregulation of the physiological stress axes may induce altered perception of bodily sensations is mainly based on the following observations:

(1) In numerous mental disorders that are accompanied by physical symptoms, altered interoception and dysregulation of physiological stress axes are reported. In detail, (a) individuals with major depression (MD) and depressive symptoms exhibit reduced IA and HEP amplitudes (Dunn et al., 2007; Terhaar et al., 2012). Concerning the activity of the HPA axis, previous research has shown differences between the melancholic and the atypical sub-type (Gold and Chrousos, 2002; O’Keane et al., 2012; Gold, 2015): on the one hand, the atypical sub-type exhibited normal basal cortisol levels and increased dexamethasone-induced feedback sensitivity (Levitan et al., 2002; O’Keane et al., 2005; O’Keane et al., 2012). On the other hand, the more frequent melancholic sub-type is characterized by hypersecretion of CRF (Nemeroff et al., 1984; Wong et al., 2000), and reduced concentration and sensitivity of CRF neurons, resulting in a blunted ACTH response to CRF administration (Gold et al., 1988; Lesch et al., 1988; Ehlert et al., 2001), elevated basal cortisol levels (Gold et al., 1988; Ehlert et al., 2001; Gold, 2015), blunted cortisol responsiveness to acute stressors and reduced feedback sensitivity as provoked by dexamethasone (Gold et al., 1995; Pariante and Lightman, 2008). (b) Patients with PD show increased IA compared to healthy individuals (Ehlers and Breuer, 1992, 1996; Ehlers et al., 1995), whereas a meta-analysis has pointed out that this difference may be due to a minority within the PD group (Willem Van der Does et al., 2000). However, PD patients exhibit an increased coupling of feedback-evoked EEG amplitude and heart rate changes than healthy individuals (Mueller et al., 2014), which has previously been shown to be an indicator of neuro-visceral connectivity (Mueller et al., 2010a). Regarding the physiological stress axes, PD patients do not differ from healthy individuals in baseline morning or diurnal cortisol release or negative feedback sensitivity (Ising et al., 2012), but they show blunted cortisol responses to psychosocial stress (Petrowski et al., 2010, 2013). In terms of SAM axis activity existing studies have yielded mixed findings. While some failed to find differences in indicators of central (e.g., low frequency heart rate variability/HRV) or peripheral sympathetic activation (α-amylase) between PD and healthy controls (Tanaka et al., 2012), others observed reduced central (McCraty et al., 2001), but increased peripheral activation (Marshall et al., 2002). However, it has been proposed that dysfunction in α2-adrenergic regulation of the ANS represents an important neurophysiological correlate of PD (Bremner et al., 1996). (c) In SDs previous studies have yielded mixed findings, which may be partially explained by the heterogeneity of symptomatology collapsed in this diagnostic category. Some studies have failed to find differences in IA between patients with SD and healthy individuals (Barsky et al., 1995; Mussgay et al., 1999; Schaefer et al., 2012), while others observed exaggerated report of bodily sensations (Bogaerts et al., 2010), resulting in an overall decrease of IA (Pollatos et al., 2011a; Weiss et al., 2014). SD patients show both alterations in baseline cortisol levels, such as the CAR (Rief et al., 1998b) or daily profile (Tak et al., 2009; Tak and Rosmalen, 2010), and aberrant cortisol responses to psychosocial stress (Janssens et al., 2012). The question, therefore, is not *whether* dysregulation of physiological stress axes, altered interoception and physical symptoms are associated, but *how* they are related and *which direction* these relationships have.
(2) In pharmacological studies, the acute administration of catecholamines (Moor et al., 2005) or cortisol (Schulz et al., 2013c) affects interoception and interoceptive signal transmission. Despite the fact that chronically altered levels of adrenergic stress hormones or cortisol are not fully comparable to an acute administration, it is unlikely that chronic alterations of these hormones do not affect interoception at all. We interpret these findings as support for the assumed direction of physiological stress axes affecting interoception in our model.

Previous studies have addressed mental disorders with physical symptoms, interoception, and indicators of only *one* physiological stress axis in the same sample. Pollatos et al. (2011a) found lower IA in patients with SD to be accompanied with decreased autonomic balance, as indicated by low/high frequency HRV ratio. In a study by Ehlers et al. (1995) higher heart rates were observed in panic patients than in healthy individuals, suggesting increased sympathetic tone in patients with PD. As both stress axes may differentially affect interoceptive processes, however, future studies are needed that include indicators of *both* stress axes at a time. Currently, there is only one study using multiple indicators of *both* physiological stress axes in the investigation of physical symptoms associated with a mental disorder (Schulz et al., 2015b). Results show an insensitivity of HEPs for attention focused on heartbeats in patients with DPD, but not in healthy individuals. This difference was associated with higher basal level of salivary α-amylase, an indicator for peripheral sympathetic activation (Chatterton et al., 1996; Nater et al., 2005). Furthermore, across DPD patients and healthy individuals there was a negative correlation between basal cortisol level and HEP amplitude (Schulz et al., 2015b). In the same sample IA did not differ between DPD and healthy control individuals (Michal et al., 2014), although differences between DPD and healthy individuals have been reported in another study (Sedeno et al., 2014).

To elucidate the direction of the relationship between dysregulated stress responses and interoception, prospective long-term observations may be required. There is a notable lack of reports on interoception in chronically stressed individuals, who already show a dysregulation of both physiological stress axes, but do not yet fulfill the diagnostic criteria for a mental disorder associated with physical symptoms. To date, there is only one study investigating the relationship of early life stress and brain activity in healthy individuals and patients with irritable bowel syndrome (IBS): Gupta et al. (2014) report altered activation of brain networks associated with pain processing and early life stress in IBS patients. The importance of chronic stress for altered interoception in IBS is further underlined by the fact that IBS patients also show altered activation of brain regions associated with interoception during stress (Elsenbruch et al., 2010a).

The question remains whether there is a direct relationship between the dysregulation of physiological stress axes and the generation of physical symptoms without the mediating effect of interoception. Based on the currently available evidence this question cannot be unequivocally answered. We do not assume a direct pathway for the following reasons: (1) There is currently no model to explain the psychobiological processes connecting the dysregulation of physiological stress axes and the generation of physical symptoms, despite a broad empirical basis showing stress axes dysregulation in mental disorders to be associated with bodily symptoms, e.g., in depression (Holsboer, 2000; Ehlert et al., 2001; Gold and Chrousos, 2002; Pariante and Lightman, 2008), FMS (Tanriverdi et al., 2007; Wingenfeld et al., 2007, 2008); IBS (Chang et al., 2009; Suarez-Hitz et al., 2012), or CFS (Cleare, 2004; Nater et al., 2008). (2) Some direct relationships between stress axes and physical symptoms are even more heterogeneous than those observed between interoception and physical symptoms, e.g., in SD. Previous studies addressing interoception in SD have either produced null findings or shown reduced IA. Meanwhile, studies investigating indicators of stress axes in SD find directly opposing findings, ranging from higher (Rief et al., 1998a; Rief and Barsky, 2005), identical (Rief and Auer, 2000) to reduced cortisol output in SD patients compared to healthy controls (Heim et al., 2000; Tak and Rosmalen, 2010; Janssens et al., 2012). A popular way of explaining these potentially conflicting findings is to emphasize the heterogeneity of symptoms collapsed into SD classification. However, an alternative explanation would be a mediating factor between stress axes and physical symptoms, i.e., altered interoceptive processes.

### Interoception and the Generation of Physical Symptoms (Path *d*)

Many mental disorders that are associated with physical symptoms are characterized by altered interoception, as discussed earlier in this paper. Among those, PD is a prominent example, where the experience of (frightening) physical symptoms is a defining diagnostic criterion. It is, therefore, not surprising that PD has had perhaps the longest standing history in the investigation of interoceptive processes, however, with very mixed results. There are reports of increased IA in panic patients (Ehlers and Breuer, 1992; Ehlers et al., 1995), but also those failing to find any differences in IA between panic patients and healthy individuals (Antony et al., 1995; Van der Does et al., 1997; Wolk et al., 2014). This mixed picture of results gave rise to the assumption that methodological differences (e.g., the precise wording of instructions for the most often employed heart-beat tracking task) between studies may account for these inconsistent findings (Ehlers et al., 1995; Ehlers and Breuer, 1996). Alternatively, the possibility of differences in IA between PD patients has been discussed (i.e., a PD subgroup high in IA; (Ehlers et al., 1995; Ehlers and Breuer, 1996; Van der Does et al., 1997; Willem Van der Does et al., 2000). Furthermore, the question arose whether IA may represent a risk factor for the development of PD symptoms. Supporting this assumption, IA was found to be higher in patients with maintained or relapsed PD and infrequent panic attacks, than in remitted patients (Ehlers, 1995). Moreover, exposure treatment did not change IA (Ehlers et al., 1995).

In contrast to PD, IA has been shown to be lower in SD (Bogaerts et al., 2010; Pollatos et al., 2011a; Weiss et al., 2014), and there also seems to be a positive association between symptom severity and IA impairment (Schaefer et al., 2012). Accordingly, reduced IA could be a risk factor for the development of SD. This hypothesis is supported in a recent study by Schaefer et al. (2014), showing that state symptom perception is reduced after cardiac IA training. Although the course of symptom severity in dependence of IA may suggest the direction of the assumed pathway, the quasi-experimental design of these studies prevents from causal inferences to be drawn. In fact, all of these studies included individuals who had already been diagnosed with a mental disorder at the time of the investigation and, therefore, a pre-morbid indicator of IA was not available. Again, to address this potential shortcoming, prospective studies in the general population or in specific high-risk groups (e.g., exposed to chronic stress) are required.

In addition to SD, reduced IA has also been observed in patients with anorexia nervosa (Pollatos et al., 2008) and obesity (Herbert and Pollatos, 2014), whereas intuitive eating is positively associated with IA (Herbert et al., 2013). The direction of the pathway between interoception and the generation of physical symptoms may depend on the type of mental disorder. As almost all studies in this field are cross-sectional and quasi-experimental in design, it is difficult to come to any conclusions on the direction of this relationship. Beyond clinically relevant eating behavior, the experimental manipulation of eating behavior using short-term food deprivation induces an increase in IA as assessed by heartbeat perception (Herbert et al., 2012) and HEP amplitudes (Schulz et al., 2015a). As some neuroendocrinological parameters, such as sympathetic tone or peptide YY output reverse from hyper- to hypo-activation in long-term fasting, one may argue that eating behavior could serve as a coping mechanism to regulate the perception of bodily sensations. The direction of the relationship between interoception and physical symptoms in eating disorders may, therefore, be reversed in disordered eating (path *d*) or even bi-directional. It needs to be acknowledged that this interpretation is based on experimentally manipulated eating behavior and its translation to pathological eating behavior (i.e., eating disorders) should be done with caution. In the interest of greater simplicity of our suggested model, we have refrained, however, from including this reversed path *d*, and acknowledge, therefore, that the current model is limited to physical symptoms and disorders that imply the passive experience of altered bodily sensations (e.g., MD, PD, SD, DPD), and does not apply to physical symptoms that are partially induced by disordered eating behavior.

### Physical Symptoms and Stress (Path *e*)

It is widely accepted that the repeated and enduring experience of physical symptoms is (probably causally) related to the experience of stress, as denoted by the term ‘symptom distress.’ The majority of reported physical ‘somatization’ symptoms involve the experience of pain (Rief et al., 2001). Chronic pain is described in the literature as severe ‘inescapable stress.’ In animal models chronic pain has been shown to induce depressive-like symptoms, which may provide a model for the co-occurrence of chronic stress, pain and depression in humans (Blackburn-Munro and Blackburn-Munro, 2001). In support of this notion, individuals with chronic pain exhibit higher perceived chronic stress and higher cortisol as assessed from hair (Van Uum et al., 2008), an indicator sensitive to chronic stress (Russell et al., 2012). Symptom distress may not necessarily have sufficient quality and intensity to be comparable to severe chronic psychosocial stress, such as caregiving to family members or harassment at working place. Nevertheless, the current model posits that the repeated exposure to physical symptoms may induce stress, thereby increasing and adding to the experience of already existing stress. In SD, symptom distress is largely affected by SD-typical forms of behavior, such as inadequate reassurance or negative interactions between patient and doctor (Rief and Broadbent, 2007). As symptom distress is associated with the automatic negative evaluation of afferent somatosensory signals (Witthoft et al., 2012), it could be hypothesized that specific cognitive styles may contribute to the positive feedback mechanism relating stress and physical symptoms. The correlational design of this study, however, does not allow for any such causal interpretations, i.e., whether automatic negative evaluation is a cause or consequence of symptom distress. In the current model, negative evaluation style may be seen as a moderator of the pathway between physical symptoms and stress. In summary, we propose that via the impact of physical symptoms on stress, the vicious circle between stress and symptoms are completed, which is characteristic of a self-maintaining positive feedback mechanism.

## Neuroendocrine Pathways

Comprehensive reviews on neural structures supporting interoceptive signal processing, and original research papers including neuroimaging data are available elsewhere (Cameron, 2001; Craig, 2002; Critchley et al., 2004; Pollatos et al., 2007a,c). In the following we provide a summary of neural pathways that are important for transmitting and processing afferent signals from the body to the brain, and which may also be involved in the experience of stress. The majority of the available literature focuses on interoception of cardiac sensations. As previously described (Schulz et al., 2013c), visceral-afferent neural signals from the cardiovascular system are relayed over the NTS, the major sensory center for visceral-afferent neural signals in the brainstem (Jänig, 2006). The NTS projects onto the parabrachial nucleus and the LC, from where hypothalamic and thalamic nuclei are reached (Cameron, 2001). Cortical structures that process visceral-afferent neural signals include the anterior cingulate cortex (ACC), the frontal cortex, the somatosensory cortex and the right insula (Cameron, 2001; Critchley et al., 2004; Pollatos et al., 2007a,c). In a dipole localization study it was demonstrated that HEPs originate from exactly these four brain areas (Pollatos et al., 2005). When focusing on brain regions that are involved in the elicitation of a stress response, specific attention is paid to the ACC and the right insula. Blood perfusion in both areas is increased after a mental arithmetic task in individuals with high self-reported stress (Wang et al., 2005). The replication of these results suggests that these areas are specifically sensitive to psychological stress in women (Wang et al., 2007). The importance of the ACC in mediating stress responses has been demonstrated in numerous studies: functional connectivity in the ACC is related to cortisol release in response to a combined dexamethasone/CRF administration in healthy individuals (Kiem et al., 2013), while ACC connectivity may be reduced in traumatic stress experience (Kennis et al., 2014). Altered insular activity can be observed in early life (Mueller et al., 2010b) and traumatic stress (Bruce et al., 2012). However, these studies may not disentangle whether the ACC and the insula are involved in the up-regulation of physiological stress axes or if their altered activity and connectivity is a result of altered afferent input from visceral organs due to the dysregulation of stress responses.

Brain regions that are sensitive to the effects of stress hormones, and play a role in interoception, involve the thalamus, and other limbic structures, such as the amygdala and hippocampus, which are important for learning and memory (see below). In particular, blood perfusion in the thalamus is reduced within a time frame of 17 min after cortisol infusion, implying a rapid, non-genomic effect of cortisol (Strelzyk et al., 2012). In contrast, when investigating a later time period, in which genomic and non-genomic mechanisms overlap, cortisol affects activity in the amygdala and hippocampus, whereas no effect on the thalamus could be observed (Lovallo et al., 2010). The thalamus is a major relay center for sensory information that processes and integrates intero- and exteroceptive signals. Cortisol may rapidly affect thalamic activity and could eventually favor the processing of interoceptive signals at the cost of others, while this effect disappears over time. Future studies should clarify if thalamic activity may be chronically altered in HPA axis dysregulation, which may play a role in the perception of physical symptoms.

At receptor level, special attention has been paid to the role of α2-adrenoceptors. As repeatedly demonstrated, early life stress (Caldji et al., 1998; Liu et al., 2000) and chronic stress (Flugge et al., 2003) may impair the expression of α2-receptors in the NTS, which plays an important role in the down-regulation of the sympathetic nervous system (Flugge, 1999) and the sensory relaying of visceral-afferent neural signals (Jänig, 2006). α2-adrenoceptors are involved in the adequate processing of visceral-afferent signals from the cardiovascular (Sved et al., 1992) and the gastrointestinal system (Myers et al., 2005). It could be argued, therefore, that the reduced density and functionality of α2-adrenoceptors under chronic stress may reduce the individual’s capacity for the adequate processing of visceral-afferent signals, which may eventually result in the generation of physical symptoms.

In SD patients there is evidence for altered activity in medullary control mechanisms for the sympathetic nervous system (Laederach-Hofmann et al., 2008), the ACC (Klug et al., 2011) and insula (Gundel et al., 2008). PD may be accompanied by reduced sensitivity of α2-adrenoceptors (Bremner et al., 1996), while reduced gray matter in the insula (Uchida et al., 2008) and altered ACC activity (Asami et al., 2008; Shin et al., 2013) could also be observed. Anorexia nervosa is characterized by changes in insular activity (Oberndorfer et al., 2013; Strigo et al., 2013). We propose that alterations in receptor sensitivity, volume, blood- flow or connectivity of the respective brain regions could be a result of stress system dysregulation and represent an important factor in altered interoception in these disorders.

Taken together, acute stress, the release of stress hormones and chronic stress may affect the processing of visceral-afferent neural signals at different brain levels, which are important for interoception, such as the NTS, the thalamus, the ACC and the insula. It remains for future research to elucidate whether certain mental disorders, which are associated with physical symptoms, can be differentiated by specific neurobiological patterns of dysregulated receptor- and brain area functioning.

## Psychobiological Mechanisms Involved in Interoception and Stress

While the present review and the proposed model focus on the role of physiological stress responses and their dysregulation for altered interoception and the generation of physical symptoms, stress responses include psychological processes that are constituent factors of the experience of stress. In the following we, therefore, briefly discuss psychological processes that may be important for interoception and stress, without making specific reference to these in the proposed model.

### Anxiety and Interoception

Individual stress responses are accompanied by mood changes that include anxiety or fear. There is indeed such an overlap of concepts, methodologies (including experimental paradigms) and physiological correlates of acute stress and anxiety that it seems all but impossible to differentiate between the two constructs (Stevens et al., 2011; Schmitz et al., 2012; Durlik et al., 2014). At least part of the literature discussed in the Section on “Acute Stress and Interoception (Path *b*)” could, therefore, also be subsumed under the title “Anxiety and Interoception.” To understand similarities and differences between both concepts, their theoretical definitions have to be evaluated. On the one hand, anxiety is a distinct emotion, which is characterized by a limited duration, an antecedent appraisal of a stimulus, and multiple components including specific affective, behavioral, cognitive, and physiological response patterns (Russell, 2003; Ekkekakis, 2013). Anxiety has a state component, i.e., a transitory condition of anxiety, and a trait component, which describes a relative stable disposition to respond to stimuli with state anxiety (Spielberger and Reheiser, 2004). On the other hand, psychological (dis-)stress is characterized as core affect (component of negative affect), which has a long duration, a variable intensity, it does not require either an antecedent appraisal or an eliciting stimulus, and is restricted to the affective component (Russell, 2003; Ekkekakis, 2013). It has to be acknowledged, however, that this definition may be limited to affective response patterns as to be expected in enduring or chronic stress, but it does not include specific physiological response patterns that are implied by chronic exposure to stress. In stress research, the definition of acute stress is commonly based on a neuro-psycho-endocrine perspective. This perspective emphasizes physiological response patterns, as earlier described, but also includes behavioral, cognitive and affective components of stress, such as increased anxiety (McEwen, 2000). In contrast to the core affect of psychological distress, in research on acute stress as induced by established laboratory stressors, an antecedent appraisal of a specific stimulus exists, which may be an additional overlap with the emotional state of anxiety. Important differences between state anxiety and acute stress would be the broader range of distinct emotions that could be elicited during acute stress (e.g., anxiety, fear, anger, etc.) that imply a more diffuse pattern of behavioral and physiological responses, and the longer duration of an acute stress response. Trait anxiety, anxiety sensitivity, and anxiety disorders are all strongly associated with interoception. For example, healthy individuals with high social anxiety show higher IA than those with low social anxiety (Stevens et al., 2011). Furthermore, anxiety sensitivity may moderate the effects of acute stress on interoception (Sturges and Goetsch, 1996). As argued by Domschke et al. (2010), anxiety sensitivity may increase the vulnerability to anxiety disorders by increasing the perceptual basis for catastrophic interpretations of physical symptoms. Although we do not explicate the role of trait anxiety or anxiety sensitivity for interoception in the current model, it may be speculated that either aspect of anxiety could potentially moderate almost all proposed variables (e.g., the probability and intensity of stress experience, physical symptoms) and pathways, e.g., between physiological stress axes and interoception (path *c*), between interoception and perception of physical symptoms (*d*) and between symptoms and stress experience (*e*).

### Attention and Interoception

In models on altered interoception in PD (Ehlers and Breuer, 1996; Willem Van der Does et al., 2000) or SD (Rief and Barsky, 2005; Rief and Broadbent, 2007) attention focused on physical symptoms plays an essential role. While there is largely agreement in the literature on attentional bias to body- and health-related sensations in patients with PD and SD, evidence on the factors or processes contributing to attentional bias (e.g., acute and chronic stress) and, therefore, physical symptom generation, is lacking. Regarding the SAM axis, the central noradrenergic system, including the LC, is of particular importance. Early life or chronic stress may decrease the expression of α2-adrenoceptors in the LC (Caldji et al., 1998; Liu et al., 2000), which are involved in the regulation of the ‘alertness’ component of attention. As described above, α2-adrenoceptors are involved in a negative feedback mechanism that down-regulates sympathetic activation and associated processes, such as alertness and vigilance. Consequently, the acute activation of α2-adrenergic receptors increases vigilance and alertness (Berridge and Foote, 1991), while their deactivation results in chronic hypervigilance and alertness (Aston-Jones et al., 1999; Usher et al., 1999). Regarding the HPA axis, the relationship between stress hormones and attentional processes may be more complex, since cortisol may affect all neurons in the entire brain. As summarized by Erickson et al. (2003), glucocorticoids facilitate focused attention at the cost of irrelevant stimuli and alter the perceptual threshold of external stimuli (Fehm-Wolfsdorf and Nagel, 1996). These effects are probably due to increased availability of norepinephrine, as caused by glucocorticoids (Irwin et al., 1986; Kvetnansky et al., 1993). Future research should clarify whether this change in perceptual threshold may also apply to interoceptive signals. Mental stress tests that have been shown to affect both stress axes, also induce increased selective attention on primary tasks at the expense of cognitive flexibility (Chajut and Algom, 2003; Plessow et al., 2011). The differential effects of stress on IA as assessed by the Schandry- and Whitehead-based heartbeat perception tasks (Schulz et al., 2013a) indicate that different facets of attention are of relevance for the impact of stress on interoception. It is plausible, for example, that focused attention on interoceptive signals is facilitated (Schandry task), while the integration of intero- and exteroceptive signals is attenuated (Whitehead task) during stress. With respect to the proposed model, attention may moderate the relationship between physiological stress axes and interoception (path *c*), between interoception and the perception of physical symptoms (*d*), and between symptoms and the experience of stress (*e*).

### The Role of Interoceptive Signal Processing in Stress Effects on Memory and Learning

Stress affects memory at acquisition, consolidation and recall. Interoceptive signal processing may contribute to some of these mechanisms (Schulz, 2015). McGaugh (2000) and Roozendaal et al. (2006) postulated the requirement of two physiological processes for the enhancement of memory consolidation after stress: (1) glucocorticoids that bind on glucocorticoid receptors within the NTS, the LC and the basolateral amygdala (BLA) and (2) peripherally circulating epinephrine, which cannot cross the blood-brain barrier, but activates visceral organs, whose afferent signals are transmitted via the vagus nerve to the NTS and LC. From these structures, via noradrenergic pathways, β-adrenergic synapses in the BLA are activated, which induces the release of cyclic adenosine monophosphate (cAMP) and cAMP-dependent protein kinase. Both substances can enhance memory consolidation. Postsynaptic efficacy of β-adrenergic synapses is increased by glucocorticoids. In animal studies it could be shown that both processes glucocorticoid secretion (Quirarte et al., 1997) and afferent signal transmission from visceral organs (Roozendaal et al., 1999) are necessary for improvement of memory consolidation. In humans, the increase of baro-afferent neural feedback induced by norepinephrine-infusion can improve long-term memory (Moor et al., 2005). Interoceptive neural signals, therefore, play an important role for the stress-induced improvement of memory consolidation. It is plausible that increased visceral-afferent input could also facilitate dysfunctional learning processes that contribute to the generation of physical symptoms, such as the formation of pain memory.

### Intuitive Decision Making

The influential somatic marker hypothesis by Damasio (1994) postulates that interoceptive signals are regularly integrated into processes of intuitive decision making. It is assumed that in every context, in which intuitive decisions are made, the outcomes of possible action alternatives are anticipated. This anticipation produces a specific visceral response to be integrated in affective responses to the expected outcome. These responses, either transmitted over somatosensory or visceral-afferent circuits, are called ‘somatic markers.’ Dunn et al. (2006) have provided a comprehensive review of the somatic marker hypothesis. In support of this theory IA positively correlates with performance in decision-making tasks (Werner et al., 2009; Wolk et al., 2014). Stress affects executive functions and thus decision making: cortisol may reduce performance in intuitive decision making tasks both in humans and rodents (van den Bos et al., 2009; Koot et al., 2013). This effect was explained by rapid, presumably non-genomic glucocorticoid effects in the orbitofrontal and insular cortex (Koot et al., 2013), which are also involved in interoceptive signal processing. Furthermore, a quadratic relationship exists between peripheral SAM axis activity and performance in decision making tasks, implying a performance increase in moderate sympathetic activation (Pabst et al., 2013). Again, since catecholamines do not cross the blood–brain barrier, afferent neural signals from visceral organs (e.g., transmitted over the vagus nerve) due to increased stimulation of interoceptors, are likely to be involved in this effect. All effects of peripheral sympathetic activation on brain functions, therefore, require neural signal transmission from visceral organs, which can be considered ‘interoceptive’ signals.

## Integration and Outlook

**FIGURE 2:**
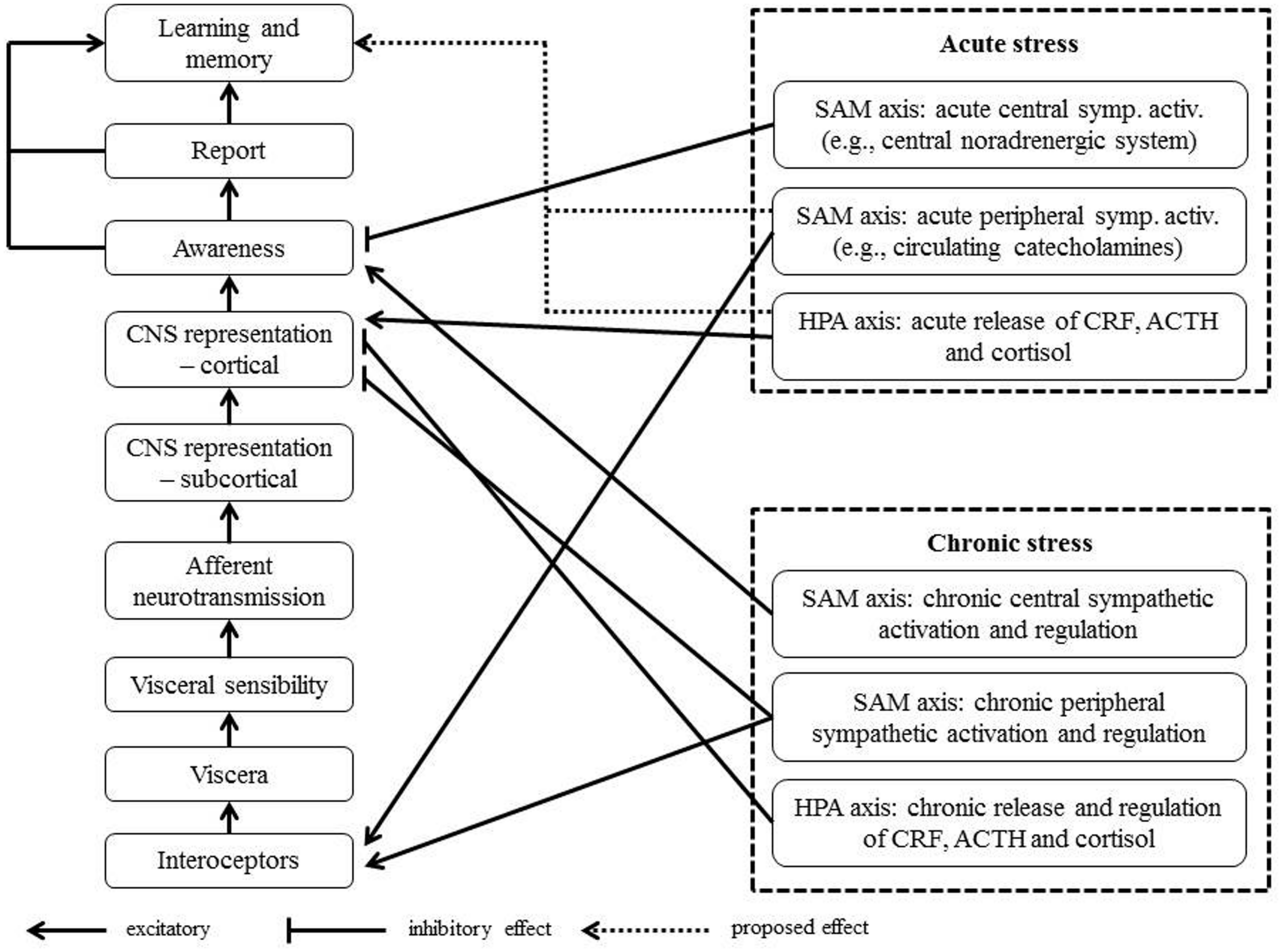
**Overview of existing findings on interoception and stress based on the model of interoception by Vaitl (1996)**.

In the earlier introduced model of interoception by Vaitl (1996) different levels of interoception were proposed. The model implies that interoception is a bottom–up process, whose effects at a low hierarchical level consecutively affect higher levels. The current review summarizes the state-of-the-art knowledge on the effects of stress (research objective 1), mainly based on research investigating afferent signals from the cardiovascular system. In **Figure 2** we integrate the reported findings on interoception and stress into a model, specifically providing links to different levels of interoception, as suggested by Vaitl (1996). In contrast to the existing model, and based on evidence of dissociations between interoceptive indicators for cortical (HEP; Schulz et al., 2013c) and brainstem processes (CMS; Schulz et al., 2010), in the current model we separated ‘CNS representation’ into cortical and sub-cortical processes. Furthermore, we focus on visceral-afferent signals and do not address the possible contribution of somatosensory information to interoception. As some conditions are associated with selective degeneration of afferent autonomic nerves (e.g., diabetes mellitus), the level ‘afferent neurotransmission’ was newly introduced into the model. Although the same mechanisms (e.g., stress axes) are involved in mediating acute stress responses and chronic stress conditions, the complex interplay between stress hormones, binding sites and feedback regulation requires their independent consideration. Acute peripheral sympathetic activation clearly increases stimulation of cardiac interoceptors (Moor et al., 2005). Given the clear physiological relationship between circulating catecholamines and stimulation of cardiac interoceptors (e.g., arterial baroreceptors), we assume that this relationship persists also in chronic elevations of catecholamine output. To our knowledge, selective effects of stress on afferent neurotransmission or sub-cortical CNS representation concerning interoception are unknown, as of to date. A first study by our group suggests that there is no effect of cortisol on the CMS (Schulz et al., 2010), possibly reflecting brainstem processing of visceral-afferent neural signals (Schulz et al., 2009a). On the one hand, acute administration of cortisol affects the cortical representation of visceral-afferent signals (Schulz et al., 2013c) as indicated by larger HEP amplitudes (when eyes are open). On the other hand, basal cortisol levels are negatively associated with HEPs (Schulz et al., 2015b). The additional finding that HEP sensitivity for attention focused on heartbeats is negatively associated with α-amylase may implicate that repeated stimulation of interoceptors could reduce sensitivity of CNS representation at a cortical level. Central sympathetic activation has particularly strong effects on attention and awareness. The overall activation of the central noradrenergic system (via α2-adrengergic antagonists) facilitates arousal and alertness, which may impair the adequate processing of visceral-afferent signals. This relationship may eventually be translated to chronic states of central sympathetic activation. It has repeatedly been demonstrated that chronic stress (Flugge, 1999) and mental disorders associated with physical symptoms are related to the dysregulation of α2-adrenoceptors (Nutt, 1989; Bremner et al., 1996). We thus assume that the chronically inadequate processing of visceral-afferent signals may reverse in increased awareness of physical sensations, which may facilitate the generation of physical symptoms. The collective effect of sympathetic activation and cortisol on learning and memory is conceptualized as ‘proposed’ effect in this model, since it has so far only been demonstrated for declarative memory (Roozendaal et al., 2004; McGaugh, 2006), although it may also play a role in the formation of memory for physical symptoms. With this model, we hope to stimulate future research on the yet unknown relationships between physiological stress systems and levels of interoceptive signal processing.

## Author Contributions

AS conceived the idea to conduct this review; AS and CV performed literature search; AS and CV interpreted the data and integrated them into the proposed framework models; and AS and CV authored the manuscript.

## Conflict of Interest Statement

The Research Office of the University of Luxembourg had no further role in conceptualization of this review, in the collection, analysis and interpretation of data, in the writing of the review, and in the decision to submit the paper for publication. The authors declare that the research was conducted in the absence of any commercial or financial relationships that could be construed as a potential conflict of interest.
